# The Complete Chloroplast Genome of Wild Rice (*Oryza minuta*) and Its Comparison to Related Species

**DOI:** 10.3389/fpls.2017.00304

**Published:** 2017-03-07

**Authors:** Sajjad Asaf, Muhammad Waqas, Abdul L. Khan, Muhammad A. Khan, Sang-Mo Kang, Qari M. Imran, Raheem Shahzad, Saqib Bilal, Byung-Wook Yun, In-Jung Lee

**Affiliations:** ^1^School of Applied Biosciences, Kyungpook National UniversityDaegu, South Korea; ^2^Department of Agriculture, Abdul Wali Khan University MardanMardan, Pakistan; ^3^Chair of Oman's Medicinal Plants and Marine Natural Products, University of NizwaNizwa, Oman

**Keywords:** wild rice (*Oryza minuta*), cp genome, repeat analysis, codon usage, phylogeny, sequence divergence, SSRs

## Abstract

*Oryza minuta*, a tetraploid wild relative of cultivated rice (family Poaceae), possesses a BBCC genome and contains genes that confer resistance to bacterial blight (BB) and white-backed (WBPH) and brown (BPH) plant hoppers. Based on the importance of this wild species, this study aimed to understand the phylogenetic relationships of *O. minuta* with other *Oryza* species through an in-depth analysis of the composition and diversity of the chloroplast (cp) genome. The analysis revealed a cp genome size of 135,094 bp with a typical quadripartite structure and consisting of a pair of inverted repeats separated by small and large single copies, 139 representative genes, and 419 randomly distributed microsatellites. The genomic organization, gene order, GC content and codon usage are similar to those of typical angiosperm cp genomes. Approximately 30 forward, 28 tandem and 20 palindromic repeats were detected in the *O*. *minuta* cp genome. Comparison of the complete *O. minuta* cp genome with another eleven *Oryza* species showed a high degree of sequence similarity and relatively high divergence of intergenic spacers. Phylogenetic analyses were conducted based on the complete genome sequence, 65 shared genes and *matK* gene showed same topologies and O. *minuta* forms a single clade with parental *O. punctata*. Thus, the complete *O*. *minuta* cp genome provides interesting insights and valuable information that can be used to identify related species and reconstruct its phylogeny.

## Introduction

The angiosperm chloroplast (cp) is a uniparentally inherited and stable structure. Accordingly, it is considered to be an informative and valuable resource for phylogenetic analysis in plants at multiple taxonomic levels (Nadachowska-Brzyska et al., 2015) compared to mitochondrial genomes (Timmis et al., 2004). Most cp genomes range from 120 to 210 kb and have a quadripartite structure that is typically composed of a small single-copy region (SSC), a large single-copy region (LSC) and a pair of inverted repeats (IRs) (Yurina and Odintsova, 1998; Wang et al., 2015). In most cases, differences in the length of the IRs determine length differences of the cp genome (Chang et al., 2006; Guisinger et al., 2011).

Previously, phylogenetic analyses have been based on sequencing one or a few loci from the chloroplast. Due to the availability of complete chloroplast sequences in public databases and advances in next-generation sequencing techniques, analyses based on the entire chloroplast genome are achievable and yield higher quality and more valuable information, which could reveal detailed insight into genomic organization (Martin et al., 2005). Indeed, examining the entire cp genome can resolve previously ambiguous phylogenetic relationships among species (Jansen et al., 2007; Moore et al., 2010). Due to availability of high-throughput sequencing technology as well as the comparatively small size and structural similarity of cp genomes, hundreds of sequencing projects in terrestrial plants have recently been reported (Wu, 2016b).

Rice is an important cereal crop that provides essential food and energy for more than half of the world's population. In addition, rice is considered a model crop for studies on cereal genomics. Two species of the genus *Oryza* (*O. sativa*, and O. *glaberrima*) are cultivated, though there are more than 20 wild species (Evenson and Gollin, 1997; Sang and Ge, 2007). Different species are categorized into 10 genome types, six are diploid (AA, BB, CC, EE, FF, and GG) (2n = 2x = 24) and the other four are allelotetraploid (BBCC, CCDD, HHJJ, and HHKK) (2n = 4x = 28) (Ge et al., 1999). About one half of the species in *Oryza* genus are allotetraploids that originated through interspecific hyberdization and genome doubling (Vaughan, 1989; Bao and Ge, 2008; Jacquemin et al., 2013). Rice (*O*. *sativa*) with an AA genome type, is one of the most important species, and it is further divided into the subspecies *japonica* and *indica*, which are distributed globally (Chang, 1976; Wambugu et al., 2015).

Because of the importance of *Oryza* as a major food crop, great attention has been given to understanding the genetic makeup and phylogeny of this genus, both within the genus and species (Guo and Ge, 2005). In plants, sequencing functional genes in cpDNA (chloroplast DNA) is helpful for resolving issues related to molecular taxonomy and phylogenetic reconstruction (Jansen et al., 2007; Moore et al., 2010; Wu and Ge, 2012), and such approaches can yield vast benefits in plant breeding and conservation strategies. Currently, 10 cp genomes belonging to Oryzeae have been published (Waters et al., 2012; Brozynska et al., 2014). Some wild *Oryza* species are better able than cultivated *Oryza* species to resist biotic and abiotic stresses and attack from insect pests. Thus, cultivated species can be improved through introgression of resistance genes from wild species (Heinrichs et al., 1985). For example, resistance traits from wild *O*. *minuta*, a tetraploid wild relative of cultivated rice, have been reported. *O*. *minuta* has a BBCC genome type and exhibits significant potential to resist blast blight, bacterial blight (BB), and white-backed plant hopper (WBPH) and brown plant hopper (BPH) diseases (Vaughan, 1994). Such diseases are damaging to the growth and yield of cultivated rice. In addition, stress tolerance genes from *O. minuta* have been successfully transferred to cultivated rice through introgression (Amante-Bordeos et al., 1992; Rahman et al., 2009). Overall, wild species such as *O. minuta* possess valuable genetic diversity that can contribute greatly to improving the growth and yield of various crops (Amante-Bordeos et al., 1992). To identify desirable genes and ensure effective conservation, it is essential to analyze phylogenetic and evolutionary relationships among species (Guo et al., 2013). Previously, it was reported that *O*. *minuta* was originated from allopolyploidization of *O*. *officinalis* (paternal) and *O*. *punctate* (meternal) (Ammiraju et al., 2010; Zou et al., 2015).

In this study, we assembled for the first time the complete chloroplast genome sequence of *O. minuta*, and performed detailed phylogenetic analyses on the basis of complete cp genome and 65 shared genes. The complete cp genome of *O. minuta*, in conjunction with previously reported cp genome sequences, will improve our understanding of *O. minuta* and the evolutionary history of genus *Oryza*. Hence, we analyzed the fully assembled cp genome of *O. minuta* and compared it to eleven closely related species: *O. australiensis* EE, *O. nivara, O. rufipogon, O*. *sativa* L. ssp. *indica, O. sativa* L. ssp. *japonica, O. barthii, O. glumipatula, O. longistaminata, O. meridionalis, O. officinalis* CC, and *O. punctata* BB.

## Materials and methods

In this study, a standard protocol for DNA extraction was used as described in detailed by Sierro et al. (2014). The extracted DNA was sequenced using an Illumina HiSeq-2000 (Illumina, San Diego, CA, USA) platform at Macrogen (Macrogen, Seoul, Korea), and the *O*. *minuta* cp genome was obtained by *de novo* assembly of the entire genome sequence via a bioinformatics pipeline (http://phyzen.com). A 400-bp paired-end library was produced according to the Illumina PE standard protocol, generating 28,110,596 bp of total reads with a 120-bp average read length. Raw reads with Phred scores of 20 or less were removed from the total PE reads using the CLC-quality trim tool, and *de novo* assembly was conducted on trimmed reads using CLC Genomics Workbench v7.0 (CLC Bio, Aarhus, Denmark) with parameters of minimum (200 to 600 bp) autonomously controlled overlap size. All contigs were then mapped and assembled against the reference cp genomes of *O. officinalis* and *O. punctata* by following a previously described method (Wu, 2016a,b). Primers were designed (Table S1) to test for correct sequence assembly. PCR amplification was performed in a total volume of 20 μl containing 1 × reaction buffer, 0.4 μl dNTPs (10 mM), 0.1 μl Taq (Solg™ h-Taq DNA Polymerase), 1 μl (10 pm/μl) primers, and 1 μl (10 ng/μl) DNA. The PCR program consisted of initial denaturation at 95°C for 5 min followed by 35 cycles of 95°C for 30 s, 65°C for 20 s and 72°C for 30 s, with a final extension step at 72°C for 5 min. After incorporation of the sequencing results, the finished cp genome was applied as a reference to map previously obtained short reads to refine the assembly based on maximum sequence coverage.

### Genome annotation and sequence architecture

The program DOGMA was used to annotate the *O*. *minuta* cp genome (Wyman et al., 2004). The annotation results were checked manually, and codon positions were adjusted by comparison to homologs from the cp genomes of *O. australiensis* and *O. sativa* ssp. *indica* in the database. All transfer RNA sequences were verified using tRNAscan-SE version 1.21 (Schattner et al., 2005) with the default settings. OGDRAW (Lohse et al., 2007) was applied to illustrate the structural features of the *O. minuta* cp genome. To examine deviations in synonymous codon usage by avoiding the influence of amino acid composition, the relative synonymous codon usage (RSCU) was determined using MEGA 6 software (Kumar et al., 2008). mVISTA software was used in the Shuffle-LAGAN mode to compare the complete variation in the *O*. *minuta* cp genome with eleven other cp genomes using the *O. minuta* annotation as a reference (Frazer et al., 2004).

### Characterization of repeat sequences and SSRs

We employed REPuter to identify repeat sequences, including palindromic, reverse, and direct repeats, within the cp genome (Kurtz et al., 2001). The following settings for repeat identification were used: (1) Hamming distance of 3; (2) 90% or greater sequence identity; (3) a minimum repeat size of 30 bp. Phobos version 3.3.12 (Leese et al., 2008) was used to detect (SSRs) within the cp genome, with the search parameters set at ten repeat units ≥10 for mononucleotides, eight repeat units ≥8 for dinucleotides, four repeat units ≥4 for trinucleotides and tetranucleotides, and three repeat units ≥3 for pentanucleotide and hexanucleotide SSRs. Tandem repeats in the *O. minuta* cp genome were identified using Tandem Repeats Finder version 4.07 b (Benson, 1999) with the default settings.

### Sequence divergence and phylogenetic analysis

Complete cp genomes as well as a separate partition using only 65 shared genes were employed to analyze the average pairwise sequence divergence for 11 *Oryza* species: *O. australiensis, O. nivara, O. rufipogon, O*. *sativa* L. ssp. *indica, O. sativa* L. ssp. *japonica, O. barthii, O. glumipatula, O. longistaminata, O. meridionalis, O. officinalis*, and *O. punctata*. Missing and ambiguous gene annotations were confirmed by comparative sequence analysis after a multiple sequence alignment and gene order comparison. These regions were aligned using MAFFT (version 7.222) (Katoh and Standley, 2013) with the default parameters. Kimura's two-parameter (K2P) model was selected to calculate pairwise sequence divergences (Kimura, 1980). To resolve the *O*. *minuta* phylogenetic position within the rice tribe (Oryzeae), 13 published cp genomes were downloaded from the NCBI database for analyses. First, multiple alignments were performed using the complete cp genomes based on the conserved structure and gene order of the chloroplast genomes (Wicke et al., 2011). Four methods were employed to construct phylogenetic trees, including Bayesian inference (BI) implemented with MrBayes 3.12 (Ronquist and Huelsenbeck, 2003), maximum parsimony (MP) with PAUP 4.0 (Swofford, 1993), and maximum likelihood (ML) and neighbor-joining (NJ) with MEGA 6 (Kumar et al., 2008) using described settings (Wu et al., 2015; Asaf et al., 2016a). In the second phylogenetic analysis, 65 shared genes from the cp genomes of 12 *Oryza* species and two *Zizania* outgroup species were aligned in ClustalX using the default settings, followed by manual adjustment to preserve reading frames. The above four phylogenetic-inference methods were used to infer trees from the 65 concatenated genes using the same settings (Wu et al., 2015; Asaf et al., 2016a).

## Results and discussion

### Chloroplast genome organization of *O. minuta*

**Figure 1:**
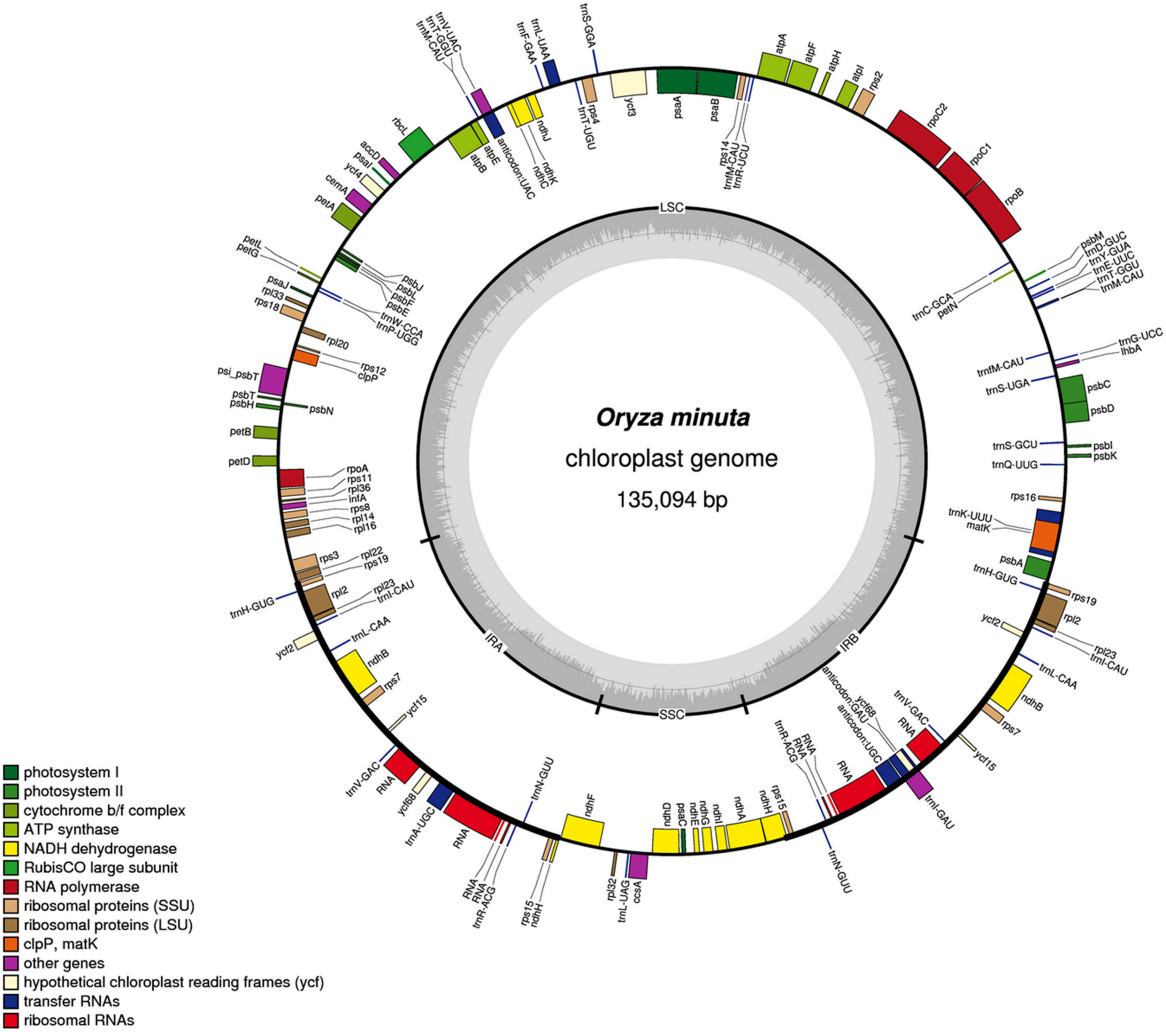
**Gene map of the ***O. minuta*** chloroplast genome**. Genes drawn inside the circle are transcribed clockwise, and those outside are transcribed counterclockwise. Genes belonging to different functional groups are color coded. The darker gray color in the inner circle corresponds to the GC content, and the lighter gray color corresponds to the AT content.

The *O. minuta* cp genome was assembled by mapping all Illumina reads to the draft cp genome sequence using CLC Genomics Workbench v7.0. A total of 1,577,251 reads were obtained, with an average length of 120 bp, for 504.211X coverage of the cp genome. The consensus sequence for a specific position was generated by assembling reads mapped with at least 875 reads per position and was used to construct the complete sequence of the *O. minuta* cp genome. The complete *O. minuta* cp genome is 135,094 bp in size (GenBank: KU179220), which is similar to the already reported cp genome sizes of related *Oryza* species and is within the range of other angiosperms (Yang et al., 2010). The cp genome possesses a typical quadripartite structure, which includes a pair of inverted repeats (IRa and IRb 20,836 bp) and separate SSC (12,446 bp) and LSC (80,974 bp) regions (Table 1, Figure 1). The GC content (39%) of the *O. minuta* cp genome is very similar to that of other *Oryza* species cp genomes (Table 1) (Wu et al., 2015). However, the GC content is unequally distributed in the *O. minuta* cp genome: it is highest in the IR regions (44.3%), moderate in the LSC regions (37.1%) and lowest in the SSC regions (33.3%). This high IR GC percentage is due to the presence of eight ribosomal RNA (rRNA) sequences in these regions. These results are similar to a previously reported high GC percentage in IR regions (Qian et al., 2013).

**Table 1:** **Summary of complete chloroplast genomes for twelve ***Oryza*** species**.

**Region**	***O. aust***	***O. min***	***O. niv***	***O. rufi***	***O. s. ind***	***O. s. jap***	***O. offi***	***O. barth***	***O. punc***	***O. meri***	***O. long***	***O. glum***
**LSC**												
Length (bp)	81,074	80,974	80,544	80,594	80,512	80,594	80,952	80,684	80,621	80,604	80,595	80,612
GC(%)	37.07	37.1	37.12	37.11	37.09	37.1	37.1	37.1	37.05	37.1	37.1	37.1
Length (%)	59.95	59.9	59.8	59.9	59.8	59.9	60	59.9	59.8	59.9	59.8	59.8
**SSC**												
Length (bp)	12,470	12,446	12,346	12,347	12,345	12,345	12,330	12,381	12,387	12,347	12,357	12,356
GC(%)	33.18	33.3	33.33	33.33	33.3	33.34	33.33	33.33	33.34	33.33	33.33	33.33
Length (%)	9.22	9.2	9.1	9.1	9.1	9.1	9.1	9.1	9.1	9.17	9.1	9.1
**IR**												
Length (bp)	20,840	20,836	20,802	20,802	20,795	20,795	20,813	20,804	20,797	20,803	20,807	20,807
GC(%)	44.33	44.3	44.35	44.35	44.3	44.3	44.3	44.3	44.4	44.4	44.33	44.33
Length (%)	15.4	15	15.4	15.4	15.4	15.4	15.4	15.4	15.4	15.4	15.4	15.4
**Total**												
GC(%)	38.95	39	39.1	39	39	39	39	39	39	39	39	39
Length (%)	135,224	135,094	134,494	134,544	134,448	134,525	134,911	134,674	134,604	134,558	134,567	134,583

*O. aust, O. australiensis; O. min, O. minuta; O. niv, O. nivara; O. rufi, O. rufipogon; O. sat. ind, O. sativa indica; O.s. jap, O. sativa japonica; O. offi, O. officinalis; O. barth, O. barthii; O. punc, O. punctate; O. meri, O. meridionalis; O. long, O. longistaminata; O. glum, O. glumipatula*.

A total of 139 genes were found in the *O. minuta* cp genome, of which 110 are unique, including 91 protein-coding genes, 40 tRNA genes, and 8 rRNA genes (Figure 1, Table 2). Of these, 11 protein-coding, four rRNA, and eight tRNA genes are duplicated in the IR regions. The LSC region comprises 62 protein-coding and 24 tRNA genes, whereas the SSC region comprises 11 protein-coding genes and one tRNA gene. The protein-coding genes present in the *O. minuta* cp genome include nine genes encoding large ribosomal proteins (*rpl2, 14, 16, 20, 22, 23, 32, 33, 36*), 12 genes encoding small ribosomal proteins (*rps2, 3, 4, 7, 8, 11, 12, 14, 15, 16, 18, 19*), *five* genes encoding photosystem I components (*psaA, B, C, I, J*), 10 genes related to photosystem II (Table 2), and six genes (*atpA, B, E, F, H, I*) encoding ATP synthase and electron transport chain components (Table 2). A similar pattern of protein-coding genes is also present in *O. sativa* (Zhang et al., 2012) and *O. glaberrima* (Wambugu et al., 2015). There are 11 intron-containing genes, 10 of which contain one intron, with only *ycf3* genes having two introns (Table S2). The *ndhA* gene has the longest intron (965 bp).

**Table 2:** **Genes in the sequenced ***O. minuta*** chloroplast genome**.

**Category**	**Group of genes**	**Name of genes**
Self-replication	Large subunit of ribosomal proteins	*rpl2, 14, 16, 20, 22, 23, 32, 33, 36*
	Small subunit of ribosomal proteins	*rps2, 3, 4, 7, 8, 11, 12, 14, 15, 16, 18, 19*
	DNA dependent RNA polymerase	*rpoA, B, C1, C2*
	rRNA genes	*RNA*
	tRNA genes	*trnA-UGC, trnC-GCA, trnD-GUC, trnE-UUC trnF-GAA, trnfM-CAU, trnG-UCC, trnH-GUG, trnI-CAU, trnI-GAU, trnK-UUU, trnL-CAA, trnL-UAA, trnL-UAG, trnM-CAU, trnN-GUU, trnP-GGG, trnP-UGG, trnQ-UUG, trnR-ACG, trnR-UCU, trnS-GCU, trnS-GGA, trnS-UGA, trnT-GGU, trnT-UGU, trnV-GAC, trnV-UAC, trnW-CCA, trnY-GUA*
Photosynthesis	Photosystem I	*psaA, B, C, I, J*
	Photosystem II	*psbA, C, D, E, F, H, I, J, K, L, M, N, T, lhbA*
	NadH oxidoreductase	*ndhA, B, C, D, E, F, G, H, I, J, K*
	Cytochrome b6/f complex	*petA, B, D, G, L, N*
	ATP synthase	*atpA, B, E, F, H, I*
	Rubisco	*rbcL*
Other genes	Translational initiation factor	*infA*
	Maturase	*matK*
	Protease	*clpP*
	Envelop membrane protein	*cemA*
	Subunit Acetyl- CoA-Carboxylate	*accD*
	c-type cytochrome synthesis gene	*ccsA*
Unknown	Conserved Open reading frames	*ycf2, 3, 4, 15, 68*

Protein, rRNAs, and tRNAs are encoded by 45.1, 6.83, and 2.2% of the entire cp genome, respectively, and the remaining 45.8% is composed of non-coding regions (Table 3). The total protein-coding sequences (CDSs) are 60,948 bp in length and consist of 91 genes encoding 20,354 codons (Tables 1, 4). The *O. minuta* cp genome codon usage frequency was determined based on tRNA and protein-coding gene sequences (Table 5). Leucine (10.7%) and cysteine (1.2%) are the maximum and minimum commonly encoded amino acids, and isoleucine, serine, glycine, arginine and alanine are encoded by 7.9, 7.5, 7.4, 6.5, and 6.1% of CDSs, respectively (Figure S1). Similar ratios for amino acids are present in previously reported cp genomes (Qian et al., 2013; Chen et al., 2015).

**Table 3:** **Comparison of coding and non-coding region sizes among twelve ***Oryza*** species**.

**Region**	***O. aust***	***O. min***	***O. niv***	***O. rufi***	***O. s. ind***	***O. s. jap***	***O. offi***	***O. barth***	***O. punc***	***O. meri***	***O. long***	***O. glum***
**PROTEIN CODING**												
Length (bp)	59,700	61,062	68,598	56,133	61,464	66,444	59,433	59,385	62,964	55,329	59,499	59,496
GC(%)	39.3	39.5	39.7	39.3	39.5	39.6	39.4	39.4	39.3	39.1	39.3	39.3
Length (%)	44.1	45.1	51	41.7	45.7	49.3	44	44	59.8	41.1	44.2	44.2
**tRNA**												
Length (bp)	2,866	3,031	2,865	2,772	2,795	2,784	2,474	2,474	3,043	3,049	2,474	2,474
GC(%)	53.2	52.1	53	52.3	53	52.9	52.7	52.7	51.7	52.6	52.7	52.7
Length (%)	2.1	2.2	2.1	2	2	2	1.83	1.83	2.2	2.2	1.83	1.83
**rRNA**												
Length (bp)	9,190	9,190	9,190	9,190	9,190	9,182	9,190	9,190	9,190	9,190	9,190	9,190
GC(%)	54.8	54.8	54.8	54.8	54.8	54.7	54.8	54.8	54.8	54.8	54.8	54.8
Length (%)	6.7	6.8	6.8	6.8	6.8	6.8	6.8	6.8	6.8	6.8	6.8	6.8
**Intergenic**	63,468	61,811	53,841	66,449	60,999	56,115	63,814	63,625	59,407	66,990	63,404	63,423
GC(%)	36	36	37	37	36	36	35	35	36	36	37	35
Length (%)	47	45.8	41	50	45.4	41.8	47.4	47.3	44.2	49.8	47.2	47.2

*O. aust, O. australiensis; O. min, O. minuta; O. niv, O. nivara; O. rufi, O. rufipogon; O. sat. ind, O. sativa indica; O.s. jap, O. sativa japonica; O. offi, O. officinalis; O. barth, O. barthii; O. punc, O. punctata; O. meri, O. meridionalis; O. long, O. longistaminata; O. glum, O. glumipatula*.

**Table 4:** **Base compositions in the ***O. minuta*** cp genome**.

	**T/U**	**C**	**A**	**G**	**Length (bp)**
Genome	30.4	19.4	30.7	19.6	135,094
LSC	31.6	18.3	31.3	18.8	80,974
SSC	30.8	17.3	35.9	16.0	12,446
IR	27.7	23.1	28	21.3	20,836
tRNA	23.5	26.1	24.3	26	3,031
rRNA	22.6	27.4	22.6	27.4	9,190
Protein-coding genes	29.9	19.5	30.5	20.0	60,948
1st position	23.27	19.0	29.3	28.2	20,354
2nd position	32.72	21.1	27.3	18.82	20,354
3rd position	37.04	14.9	31.66	16.5	20,354

**Table 5:** **The codon–anticodon recognition pattern and codon usage for the ***O. minuta*** chloroplast genome**.

**Amino acid**	**Codon**	**No**	**RSCU**	**tRNA**	**Amino acid**	**Codon**	**No**	**RSCU**	**tRNA**
Phe	UUU	733	1.28		Ala	GCA	378	1.18	*trnA-UGC*
Phe	UUC	407	0.7	*trnF-GAA*	Ala	GCG	160	0.5	
Leu	UUA	710	1.9	*trnL-UAA tRNA*	Tyr	UAU	567	1.5	
Leu	UUG	402	1.1	*trnL-CAA tRNA*	Tyr	UAC	176	0.47	*trnY-GUA tRNA*
Leu	CUU	473	1.29		Stop	UAG	22	0.74	
Leu	CUC	165	0.4		Stop	UGA	24	0.80	
Leu	CUA	319	0.87	*trnL-UAG tRNA*	Stop	UAA	43	1.44	
Leu	CUG	120	0.32		His	CAU	351	1.49	
Ile	AUU	820	1.51		His	CAC	119	0.50	*trnH-GUG tRNA*
Ile	AUC	323	0.5	*trnI-GAU tRNA*	Gln	CAA	521	1.53	*trnQ-UUG tRNA*
Ile	AUA	485	0.89		Gln	CAG	167	0.49	
Met	AUG	499	1	*trnM-CAU tRNA*	Asn	AAU	579	1.44	
Val	GUU	450	1.50		Asn	AAC	222	0.55	*trnQ-UUG tRNA*
Val	GUC	140	0.46	*trnV-GAC tRNA*	Lys	AAA	752	1.44	*trnK-UUU tRNA*
Val	GUA	442	1.47	*trnV-UAC tRNA*	Lys	AAG	291	0.55	
Val	GUG	163	0.54		Asp	GAU	558	1.55	
Ser	UCU	383	1.56		Asp	GAC	159	0.44	*trnD-GUC tRNA*
Ser	UCC	304	1.23	*trnS-GGA tRNA*	Glu	GAA	764	1.48	*trnE-UUC tRNA*
Ser	UCA	254	1.03	*trnS-UGA tRNA*	Glu	GAG	267	0.51	
Ser	UCG	120	0.48		Cys	UGU	177	1.50	
Ser	AGU	306	1.24		Cys	UGC	58	0.49	
Ser	AGC	105	0.42	*trnS-GCU tRNA*	Trp	UGG	356	1	*trnW-CCA tRNA*
Pro	CCU	351	1.59		Arg	CGU	290	1.36	*trnR-ACG tRNA*
Pro	CCC	190	0.86		Arg	CGC	110	0.51	
Pro	CCA	236	1.07	*trnP-UGG tRNA*	Arg	CGA	264	1.24	
Pro	CCG	105	0.47		Arg	CGG	102	0.48	
Thr	ACU	455	1.68		Arg	AGA	377	1.77	*trnR-UCU tRNA*
Thr	ACC	208	0.76	*trnT-GGU tRNA*	Arg	AGG	131	0.61	
Thr	ACA	294	1.08	*trnT-UGU tRNA*	Gly	GGU	493	1.28	
Thr	ACG	124	0.45		Gly	GGC	161	0.42	
Ala	GCU	553	1.72		Gly	GGA	582	1.52	*trnG-UCC tRNA*
Ala	GCC	189	0.59		Gly	GGG	295	0.77	

Among these, the maximum and minimum codons used are ATT (820), encoding isoleucine, and TTG and ATT (1, 1), encoding methionine. The AT content is 52.5, 60.0, and 68.7% at the 1st, 2nd, and 3rd codon positions, respectively, within CDS regions (Table 4). The preference for a high AT content at the 3rd codon position is similar to the A and T concentrations reported in various terrestrial plant cp genomes (Morton, 1998; Nie et al., 2012; Qian et al., 2013). In total, 42.65 and 57% of all types of preferred synonymous codons (RSCU>1) ending with A and U and C and G, respectively, were found. Non-preferred synonymous codons (RSCU <1) are 42.40 and 57.50% for C and G and A and U. Usage of the start codon AUG and UGG, the latter encoding tryptophan, has no bias (RSCU = 1) (Table 5).

### Repeat analysis

**Figure 2:**
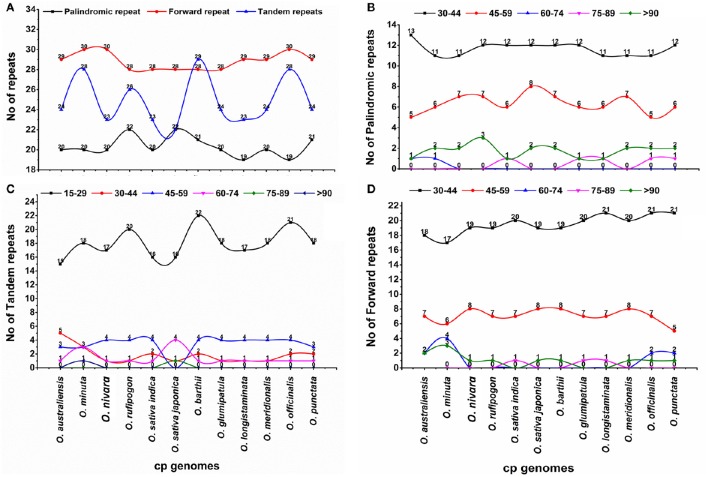
**Analysis of repeated sequences in twelve ***Oryza*** chloroplast genomes. (A)** Total of three repeat types; **(B)** frequency of the palindromic repeat by length; **(C)** frequency of the tandem repeat by length; **(D)** frequency of forward repeat by length.

Repeat sequences, which play a role in genome rearrangements, are very helpful in phylogenetic studies (Cavalier-Smith, 2002; Nie et al., 2012). Furthermore, analyses of various cp genomes revealed that repeat sequences are essential to induce *indels* and substitutions (Yi et al., 2013). Repeat analysis of the *O. minuta* cp genome showed 20 palindromic repeats, 30 forward repeats, and 28 tandem repeats (Figure 2A). Among these, 17 forward repeats are 30–44 bp in length, with only three tandem repeats of the same length and 18 15–29 bp in length (Figures 2A–D). Similarly, 11 palindromic repeats are 30–44 bp, and 6 repeats are 45–59 bp in length (Figure 2B). Overall, 78 repeats were found in the *O. minuta* cp genome. Similarly, 73, 73, 76, 71 72, 78, 72, 71, 73, 77, and 74 repeat pairs were found in previously reported *O. australiensis, O. nivara, O. rufipogon, O*. *sativa* L. ssp. *indica, O. sativa* L. ssp. *japonica, O. barthii, O. glumipatula, O. longistaminata, O. meridionalis, O. officinalis* and *O. punctata* genomes, respectively (Figure 2A). This suggests that *O. minuta* is more similar to *O. barthii* and *O. officinalis* in terms of repeats. Approximately 29.4% of these repeats are distributed in protein-coding regions. Previous reports suggest that sequence variation and genome rearrangement occur due to the slipped-strand mispairing and improper recombination of these repeat sequences (Cavalier-Smith, 2002; Asano et al., 2004; Timme et al., 2007). Furthermore, the presence of these repeats indicates that the locus is a crucial hotspot for genome reconfiguration (Gao et al., 2009; Nie et al., 2012). Additionally, these repeats are an informative source for developing genetic markers for phylogenetic and population studies (Nie et al., 2012).

### SSR analysis

**Figure 3:**
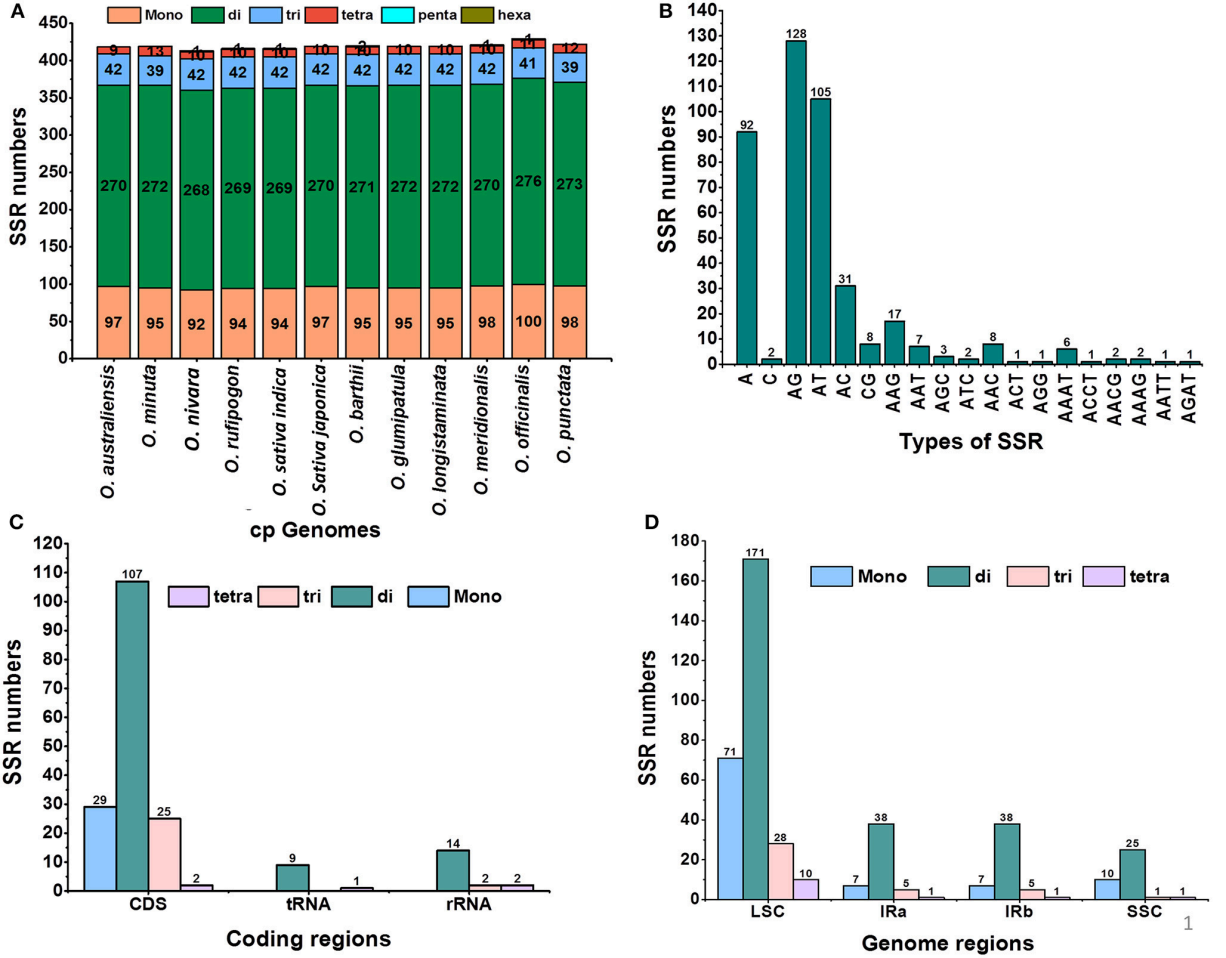
**Analysis of simple sequence repeats (SSRs) in twelve ***Oryza*** chloroplast genomes. (A)** Number of different SSR types detected in twelve genomes; **(B)** frequency of identified SSR motifs in different repeat class types; **(C)** frequency of identified SSRs in coding regions; **(D)** frequency of identified SSRs in LSC, SSC and IR regions.

Simple sequence repeats (SSRs), or microsatellites, are repeating sequences of typically 1–6 bp that are distributed throughout the genome. In this study, we detected perfect SSRs in *O*. *minuta* together with 11 other *Oryza* species cp genomes (Figure 3A). Certain parameters were set because SSRs of 10 bp or longer are prone to slipped-strand mispairing, which is believed to be the main mechanism for SSR polymorphisms (Rose and Falush, 1998; Raubeson et al., 2007; Huotari and Korpelainen, 2012). A total of 419 perfect microsatellites were found in the *O. minuta* cp genome (Figure 3A). Similarly, 418, 413, 416, 416, 419, 420, 419, 419, 421, 429, and 422 SSRs were detected in *O. australiensis, O. nivara, O. rufipogon, O*. *sativa* L. ssp. *indica, O. sativa* L. ssp. *japonica, O. barthii, O. glumipatula, O. longistaminata, O. meridionalis, O. officinalis* and *O. punctata*, respectively (Figure 3A). The majority of SSRs in these cp genomes possess a dinucleotide repeat motif, varying in quantity from 269 in *O. sativa* ssp. *indica* to 276 in *O. officinalis*. Mononucleotide SSRs are the second most common, ranging from 92 in *O. nivara* to 100 in *O. officinalis*. Using our search criterion, only one pentanucleotide SSR was found in *O. nivara, O. rufipogon, O. indica* and *O. officinalis* (Figure 3A). In *O. minuta*, most mononucleotide SSRs are A (97%) and T (2.12.30%) motifs, with the majority of dinucleotide SSRs being A/G (47.05%) and A/T (38.60%) motifs (Figure 3B). Approximately 62% of SSRs are located in non-coding regions; approximately 4.3% are present in rRNA sequences and 2.3% in tRNA genes (Figure 3C). Further analysis revealed that approximately 66.82% of SSRs occur in the LSC region, whereas 24.34 and 8.83% were found in IR and SSC regions, respectively (Figure 3D). These results are similar to previous reports that SSRs are unevenly distributed in cp genomes, and the findings might provide more information for selecting effective molecular markers for detecting intra- and interspecific polymorphisms (Powell et al., 1995a,b; Provan et al., 1997; Pauwels et al., 2012). Furthermore, most mononucleotides and dinucleotides are composed of A and T, which may contribute to bias in base composition, consistent with other cp genomes (Li et al., 2013). Our findings are comparable to previous reports that SSRs found in cp genome are generally composed of polythymine (polyT) or polyadenine (polyA) repeats and infrequently contain tandem cytosine (C) and guanine (G) repeats (Kuang et al., 2011). Therefore, these SSRs identified contribute to the AT richness of the *O. minuta* cp genome, as previously reported for various species (Kuang et al., 2011; Chen et al., 2015).

### Structural and sequence comparisons of cp genomes in *Oryza*

Eleven complete cp genomes within the *Oryza* genus (*O. australiensis, O. nivara, O. rufipogon, O. sativa* L. ssp. *indica*, O. *sativa* L. ssp. *japonica, O. barthii, O. glumipatula, O. longistaminata, O. meridionalis, O. officinalis*, and *O. punctata*) were selected for comparison with that of O. minuta (135,094 bp). *O. australiensis* has the largest genome, and this difference is mostly attributed to variation in the length of the LSC region (Table 1). Analysis of genes with known functions showed that *O. minuta* shares 65 protein-coding genes with eleven other *Oryza* species. The number of unique genes found in *O. australiensis, O. nivara, O. rufipogon, O. sativa* L. ssp. *indica, O. sativa* L. ssp. *japonica, O. barthii, O. glumipatula, O. longistaminata, O. meridionalis, O. officinalis*, and *O. punctata* was 110, 100, 101, 108, 80, 104, 104, 104, 100, 104 and 114, respectively (Table S3). Furthermore, the *O. minuta* cp genome has a gene content and organization that are similar to other *Oryza* species and members of Poaceae (Wicke et al., 2011); however, as for other grasses, it lacks a ycf1 gene, and the accD gene is a truncated pseudogene. Because these genes are essential for the survival of photosynthetic plants (Drescher et al., 2000; Kode et al., 2005), they were most likely functionally transferred to the nucleus or functionally replaced by a eukaryotic gene, as observed for the accD plastid gene in other plant families (Babiychuk et al., 2011; Rousseau-Gueutin et al., 2011).

**Figure 4:**
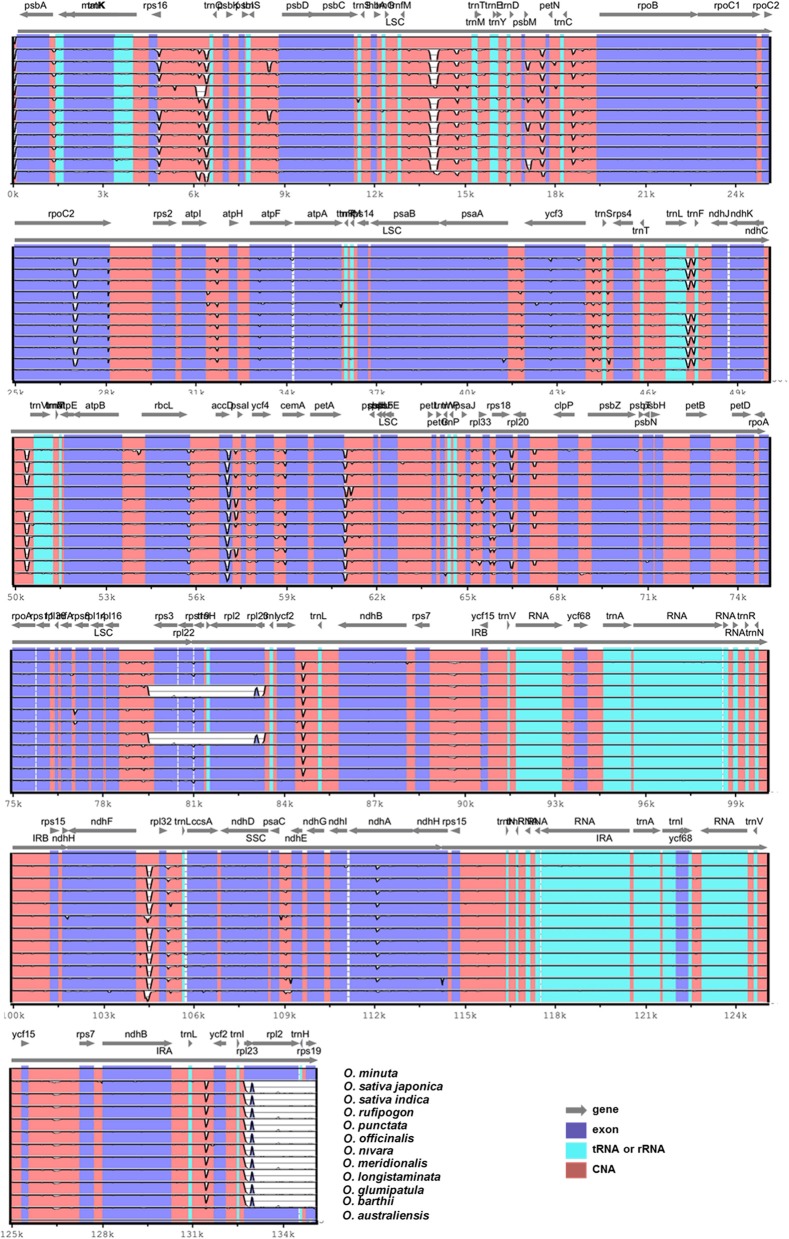
**Alignment of twelve chloroplast genome sequences**. VISTA-based identity plot showing sequence identity among twelve *Oryza* species using *O. minuta* as a reference. The thick black line shows the inverted repeats (IRs) in the chloroplast genomes.

Pairwise cp genomic alignment between *O*. *minuta* and the 11 other genomes showed a high degree of synteny. The *O. minuta* cp genome annotation was used as a reference for plotting the overall sequence identity of the cp genomes of the 11 *Oryza* species in mVISTA (Figure 4), and the results revealed high sequence identity with all 11 *Oryza* species. However, except for *O. australiensis*, relatively lower identity was also observed with these species in various comparable genomic regions, particularly the *rps3, rpl22, rpl23, rpl2*, and *rps19* regions (Figure 4). In addition, the LSC and SSC regions show less similarity than the two IR regions in all *Oryza* species. In addition, non-coding regions exhibit greater divergence than coding regions. These highly divergent regions include *rbcL, rps16-trnQ, trnfM-trnM, psbM-petN, rpoC2, atpI-atpH, ndhA rpl33, petA-psbJ, ccsA, ndhF-rpl32*, and *ycf3*. Similar results related to these genes were also reported by Qian et al. (2013). Our results also confirm similar differences among various coding regions in the analyzed species, as suggested by Kumar et al. (2009).

**Figure 5:**
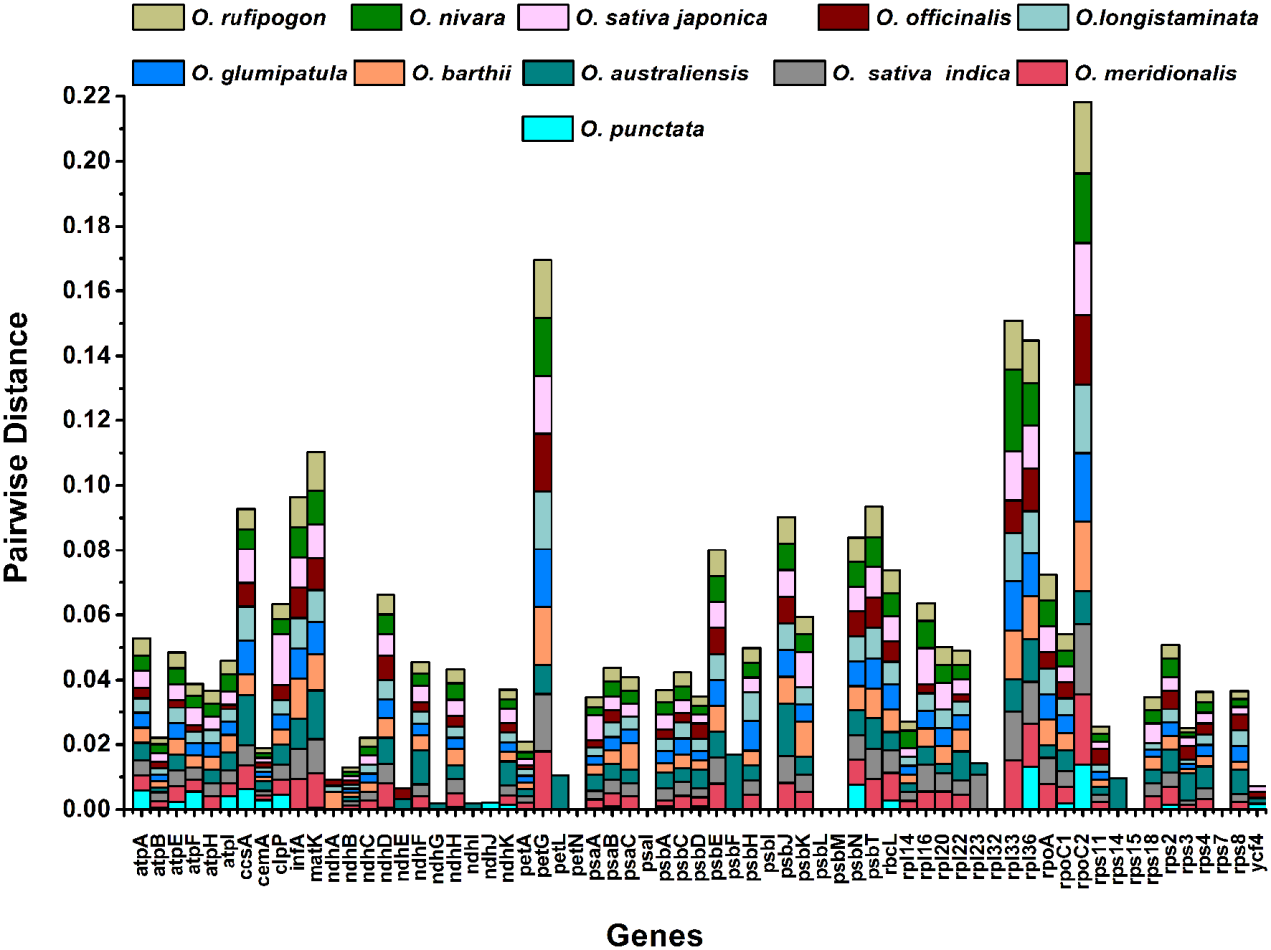
**Pairwise sequence distances of ***Oryza minuta*** genes with ***O. australiensis***, ***O. nivara***, ***O. rufipogon***, ***O. sativa*** L. ssp. ***indica***, ***O. sativa*** L. ssp. ***japonica***, ***O. barthii***, ***O. glumipatula***, ***O. longistaminata***, ***O. meridionalis***, ***O. officinalis***, and ***O. punctata*****.

We compared the cp genomes and calculated the average pairwise sequence divergence among the 12 species (Table S4). Of these, the *O*. *minuta* genome has 0.005 average sequence divergence, and high divergence was found for *O. australiensis* (0.00725); *O*. *officinalis* has the lowest average sequence divergence (0.0044). Furthermore, the twelve most divergent genes among these genomes are *petG, matK, infA, ccsA, rpoC2, clcP, psbE, rbcL, psbN, rps18, rpl36*, and *ndhF*. The highest average sequence distance was found for *rpoC2* (0.01983), followed by *petG* (0.0154) (Figure 5). Both these genes are located in LSC regions and display a trend toward more rapid evolution.

### IR contraction and expansion

**Figure 6:**
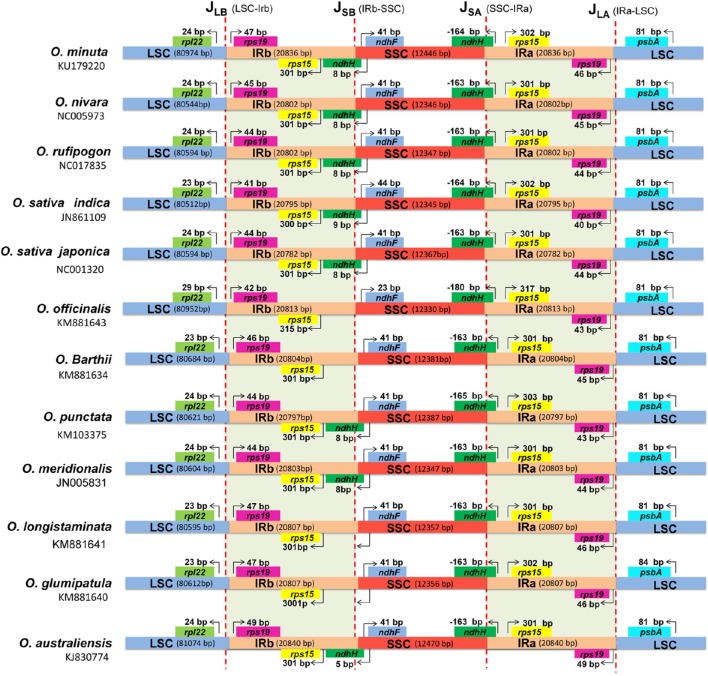
**Comparison of border distances between adjacent genes and junctions of LSC, SSC, and two IR regions among chloroplast genomes of twelve ***Oryza*** species**. Boxes above or below the main line indicate the adjacent border genes. The figure is not to scale with regard to sequence length and only shows relative changes at or near IR/SC borders.

Expansion and contraction at the borders of IR regions are the main reason for size variations in the cp genome and play a vital role in its evolution (Raubeson et al., 2007; Wang et al., 2008; Yang et al., 2010, 2014). A detailed comparison on four junctions (J_LA_, J_LB_, J_SA_, and J_SB_) between the two IRs (IRa and IRb) and the two single-copy regions (LSC and SSC) was performed among *O. australiensis, O. nivara, O. rufipogon, O*. *sativa* L. ssp. *indica, O. sativa* L. ssp. *japonica, O. barthii, O. glumipatula, O. longistaminata, O. meridionalis, O. officinalis* and *O. punctata* with regard to *O. minuta* by carefully analyzing the exact IR border positions and adjacent genes (Figure 6). Despite the similar length of the *O*. *minuta* IR region with the other eleven *Oryza* species, from 20,836 bp to 20,840 bp, some IR expansion and contraction was observed. J_LA_ is located between *rps19* and *psbA*, and variation in distances between *rps19* and J_LA_ range from 40 to 49 bp across all species; the distance in *O*. *minuta* is 46 bp. The distance between *psbA* and J_LA_ is 81 bp in *O*. *minuta*, which is similar to the other genomes (81 bp). The distance between *rpl22* and J_LB_ varies from 23 bp to 29 bp. In *O. minuta*, 1-bp variations exist in the J_SA_ border region compared to the other cp genomes. The *ndhH* gene traverses the SSC and IRa regions, with approximately 164 bp located in the IR region for *O. minuta*. Furthermore, there are 16-bp variations observed compared with *O. officinalis* for *ndhF, ndhH* and *rps15* in the SSC and IRb regions, located 41 bp, 164 bp and 302 bp from the J_SB_ and J_SA_ border regions, respectively.

### Phylogenetic analysis

**Figure 7:**
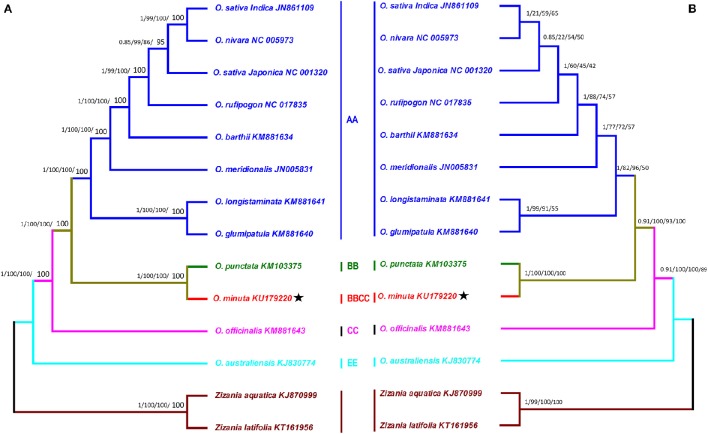
**Phylogenetic trees were constructed for 14 species from the rice tribe using different methods, and two Bayesian trees are shown for data sets of the entire genome sequence and 65 shared genes. (A)** The entire genome sequence data set **(B)**. The data set of 65 shared genes. Each data set was used with four different methods, Bayesian inference (BI), maximum parsimony (MP), maximum likelihood (ML) and neighbor-joining (NJ). Numbers above the branches are the posterior probabilities of BI and bootstrap values of MP, ML, and NJ, respectively. Stars represent positions for *O. minuta* (KU179220) in the two trees.

The *Oryza* genus is composed of 23 species distributed in different regions of America, Africa, Asia, and Australia (Ge et al., 1999). Continued efforts have expanded our ability to differentiate among and to understand the genomic structure and phylogenetic relationships of rice species (Khush, 1997). Taxonomy and phylogeny of the rice genus have been extensively investigated at genus level (Ge et al., 1999; Zhu and Ge, 2005; Jacquemin et al., 2013). Previous evolutionary relationships among different rice genomes and species were estimated by nuclear and chloroplast DNA restriction fragment-length polymorphisms (Ge et al., 1999; Zou et al., 2015), but complete genome sequencing provides more detailed insight (Wambugu et al., 2015; Wu et al., 2015; Asaf et al., 2016b). In this regard, *O. minuta* has been poorly investigated. In this study, the phylogenetic position of *O. minuta* within *Oryza* was established by utilizing complete cp genomes and 65 shared genes among 12 *Oryza* members (Figures 7A,B). Two species, *Zizania aquatic* and *Zizania latifolia* were set as outgroups. Phylogenetic analysis using Bayesian inference (BI), maximum parsimony (MP), maximum likelihood (ML) and neighbor-joining (NJ) methods were performed. The results showed same phylogenetic signals for the complete cp genomes and 65 shared genes of *O. minuta*. The complete genome sequences (Table S5) and 65 shared genes (Tables S3, S6) from all species generated phylogenetic trees with same topologies (Figures 7A,B). In these phylogenetic trees based on the entire genome data set and 65 shared genes, *O. minuta* formed a single clade with *O. punctata*, with high BI and bootstrap support using four different methods (Figures 7A,B). Furthermore, the tree topology confirmed the relationship inferred from the phylogenetic work conducted by Ge et al. (1999) and Zou et al. (2015). This position of *O. minuta* confirms the previously published phylogeny described by Ge et al. (1999). Ge et al. (1999) reported that *O. minuta* BBCC shares a clade with *O. punctata* BB with regard to *Adh1*, whereas it forms a clade with *O. officinalis* CC in the *Adh2* phylogenetic analysis. Similar resuls was suggested by Zou et al. (2015), whereby phylogenetic analysis of the four nuclear loci and three meternally interited chloroplast fragments from different *Oryza* species grouped *O*. *minuta* in a clade with maternal parent *O*. *punctata* BB (Zou et al., 2015). As the phylogenetic tree based on the *mat*K gene represents the maternal genealogy of rice species, which can offer an opportunity to identify maternal parents of allotetraploid species, we performed an additional phylogenetic analysis of *O. minuta* using the *mat*K gene from related species (Figure S2). The results revealed a single clade for *O. minuta* with parental *O. punctata*. Similar results was also suggested by Ge et al. (1999), whereby phylogenetic analysis of the *mat*K gene from different *Oryza* species grouped *O. minuta* in a clade with the maternal parent *O. punctata* BB instead of *O. officinalis* CC. Furthermore, the result suggests that there is no conflict between the entire genome data set and 65 shared genes of these cp genomes.

## Conclusion

This study reports the first complete chloroplast genome sequence of *O. minuta* (135,094 bp). The structure and organization of this genome is very similar to previously reported cp genomes from the tribe Oryzeae. The location and distribution of repeat sequences was detected, and sequence divergences among cp genomes and 65 shared genes were identified with related species. No major structural rearrangement of *Oryza* species cp genomes was observed. Phylogenetic analyses showed that data sets based on the entire genome and 65 shared genes generate trees with same topologies regarding the placement of *O. minuta*. These findings provide a valuable analysis of the complete cp genome of *O*. *minuta*, which can be used to identify species and clarify taxonomic questions.

## Author contributions

All authors listed, have made substantial, direct and intellectual contribution to the work, and approved it for publication.

## Funding

All the research work was financially supported by National Research Foundation of Korea (NRF), Ministry of Science, ICT and Future-Planning through Basic-Science Research Program (2014R1A1A1004918).

### Conflict of interest statement

The authors declare that the research was conducted in the absence of any commercial or financial relationships that could be construed as a potential conflict of interest.

## Supplementary material

Table S1 **Primers used for gap closure in *O. minuta***.

Table S2 **Genes with introns in the *O. minuta* chloroplast genome and the lengths of exons and introns**.

Table S3 **List of shared and unique genes among 12 *Oryza* cp genomes**.

Table S4 **Average pairwise sequence distance of *O*. *minuta* with the cp genome of 11 *Oryza* species**.

Table S5 **The full alignment of complete cp genomes from the 14 species examined (NEXUS format)**.

Table S6 **The alignment of 65 shared genes from the 14 species cp genomes examined (NEXUS format)**.

Figure S1 **Amino acid frequencies in *O. minuta* cp protein-coding sequences**. The frequencies of amino acids were calculated for all 97 protein-coding genes from start to stop codon.

Figure S2 **A phylogenetic tree was constructed based on 14 species from the rice tribe using different methods**. *mat*K gene sequence data were used with maximum parsimony (MP), maximum likelihood (ML) and neighbor-joining (NJ) approaches. Numbers above the branches are the bootstrap values of MP, ML, and NJ, respectively. Stars represent position for *O. minuta* (KU179220).
