# Supplementary material for: The Complete Chloroplast Genome of Wild Rice (Oryza minuta) and Its Comparison to Related Species
Source: Front Plant Sci. 2017 Mar 7;8:304. doi: 10.3389/fpls.2017.00304 (PMC5339285; doi:10.3389/fpls.2017.00304)
Supplement: Table S1 — Primers used for gap closure in O. minuta. [file DataSheet1.docx]

**S1 Table. Primers used for gap closing and sequencing verification in *O. minuta***

| Number | Primers | Sequence | Information |
| --- | --- | --- | --- |
| 1 | 1103-F | CACAGGCTTGTACTTTCGCG | Gap Closing |
|  | 1397-R | CTCGAACCCGGAACTAGTCG |  |
| 2 | 104495-F | AAGCAACTCCCTTGTTTCGT | Gap Closing |
|  | 105011-R | TGCCGCAAATCAATCCATCT |  |
| 3 | 5040-F | ACCTAAAAGAAGCAACTCCA | Gap Closing |
|  | 5820-R | ACTATTGCAATGAAAAGAAA |  |
| 4 | 27160-F | GCGATGAATCAGGTCCGAC | Gap Closing |
|  | 28120-R | GGCCTTGAACTTCTACTTTC |  |
| 5 | 6000-F | AGACCCTATCGTTTATCCTT | Gap Closing |
|  | 6640-R | ATGGATCAACAACCAAACC |  |
| 6 | 14780-F | GGACCCGTCCACTTAGATG | Gap Closing |
|  | 15630-R | GAATAGGAAAACTCGCTATT |  |
| 7 | 47920-F | TAGATAAAGCTCTAAATAGA | Gap Closing |
|  | 48168-R | GGAATTAATTAGAATGTAAT |  |
