# Supplementary material for: The Complete Chloroplast Genome of Wild Rice (Oryza minuta) and Its Comparison to Related Species
Source: Front Plant Sci. 2017 Mar 7;8:304. doi: 10.3389/fpls.2017.00304 (PMC5339285; doi:10.3389/fpls.2017.00304)
Supplement: Table S2 — Genes with introns in the O. minuta chloroplast genome and the lengths of exons and introns. [file DataSheet2.docx]

**S2 Table**. **The genes with introns in the *O. minuta* chloroplast genome and the length of exons and introns.**

| **Gene** | **Location** | **Exon I (bp)** | **Intron 1 (bp)** | **Exon II (bp)** | **Intron II (bp)** | **Exon III (bp)** |
| --- | --- | --- | --- | --- | --- | --- |
| *atpF* | LSC | 159 | 809 | 405 |  |  |
| *ndhA* | LSC | 549 | 965 | 540 |  |  |
| *ndhB* | IRb | 777 | 712 | 756 |  |  |
| *ndhB* | IRa | 777 | 712 | 756 |  |  |
| *rpl2* | IRa | 393 | 660 | 432 |  |  |
| *rpl2* | IRb | 393 | 660 | 432 |  |  |
| *ycf2* | IRb | 195 | 195 | 156 |  |  |
| *ycf2* | IRa | 195 | 195 | 156 |  |  |
| *ycf3* | LSC | 132 | 737 | 228 | 719 | 159 |
| *ycf68* | IRb | 288 | 1 | 114 |  |  |
| *ycf68* | IRa | 111 | 1 | 285 |  |  |
