# Supplementary figures and images for: The Complete Chloroplast Genome of Wild Rice (Oryza minuta) and Its Comparison to Related Species
Source: Front Plant Sci. 2017 Mar 7;8:304. doi: 10.3389/fpls.2017.00304 (PMC5339285; doi:10.3389/fpls.2017.00304)

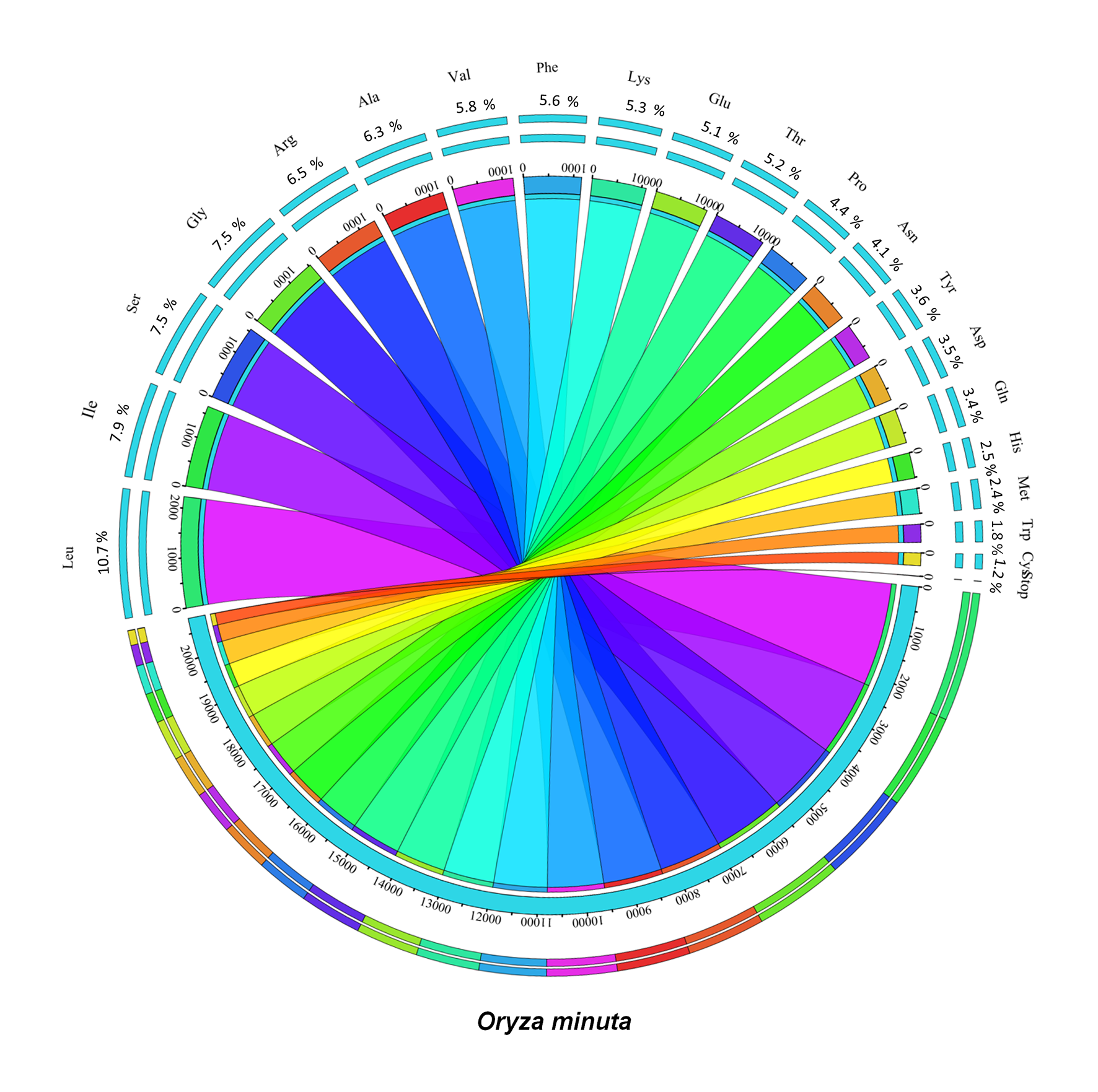

Supplement: Figure S1 — Amino acid frequencies in O. minuta cp protein-coding sequences. The frequencies of amino acids were calculated for all 97 protein-coding genes from start to stop codon. [file Image1.tif]

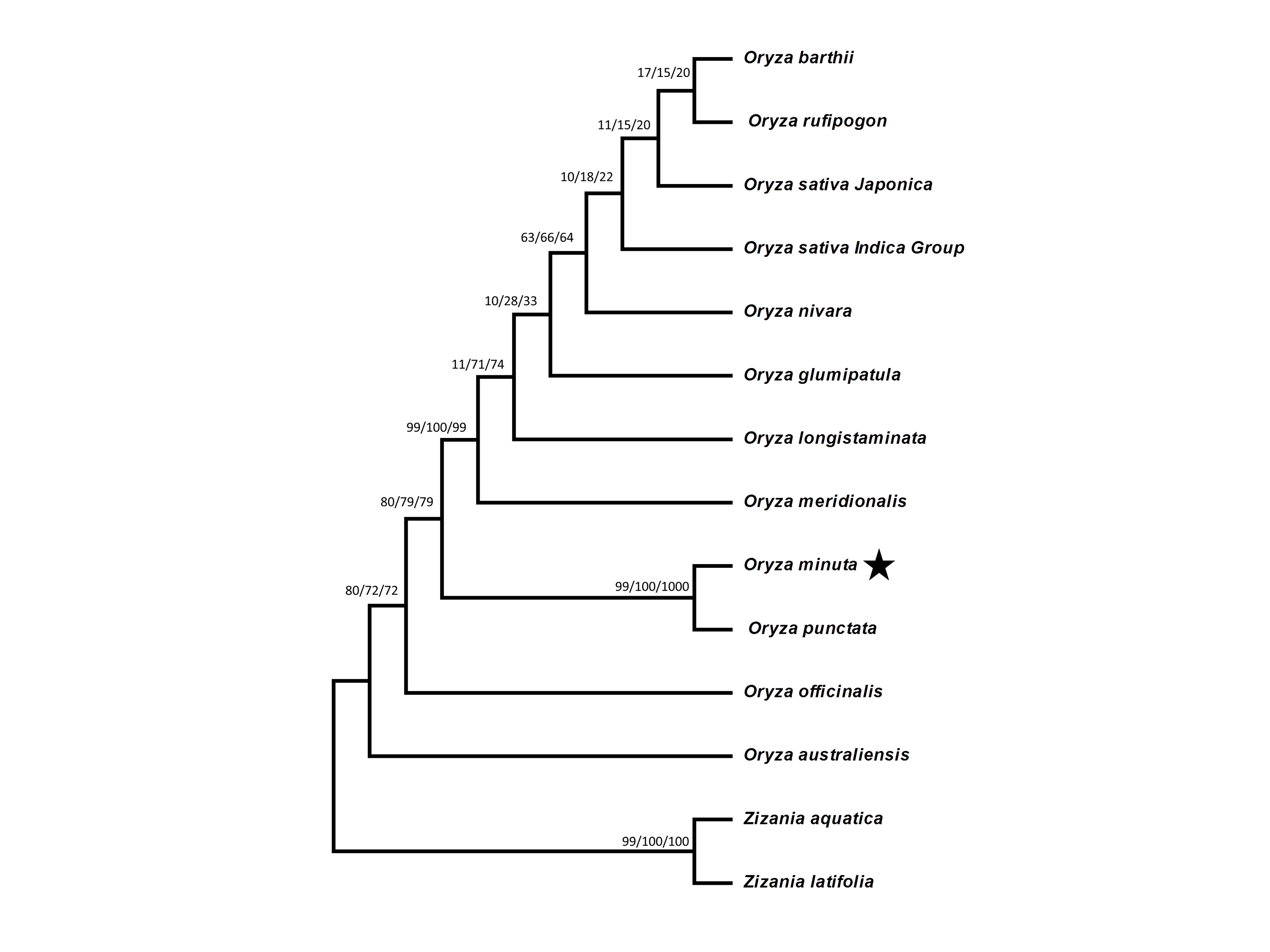

Supplement: Figure S2 — A phylogenetic tree was constructed based on 14 species from the rice tribe using different methods. matK gene sequence data were used with maximum parsimony (MP), maximum likelihood (ML) and neighbor-joining (NJ) approaches. Numbers above the branches are the bootstrap values of MP, ML, and NJ, respectively. Stars represent position for O. minuta (KU179220). [file Image2.tif]
